# Elucidating the Therapeutic Utility of Olaparib in Sulfatide-Induced Human Astrocyte Toxicity and Neuroinflammation

**DOI:** 10.1007/s11481-023-10092-9

**Published:** 2023-11-04

**Authors:** Marianna Mekhaeil, Melissa Jane Conroy, Kumlesh Kumar Dev

**Affiliations:** 1 Drug Development Research Group, Department of Physiology, School of Medicine, Trinity College Dublin, Dublin, Dublin 2 Ireland; 2 Cancer Immunology Research Group, Department of Physiology, School of Medicine, Trinity College Dublin, Dublin, Dublin 2 Ireland

**Keywords:** Astrocyte, Metachromatic leukodystrophy, Olaparib, Inflammation, Calcium

## Abstract

Metachromatic leukodystrophy (MLD) is a severe demyelinating, autosomal recessive genetic leukodystrophy, with no curative treatment. The disease is underpinned by mutations in the arylsulfatase A gene (ARSA), resulting in deficient activity of this lysosomal enzyme, and consequential accumulation of galactosylceramide-3-O-sulfate (sulfatide) in the brain. Most of the effects in the brain have been attributed to the accumulation of sulfatides in oligodendrocytes and their cell damage. In contrast, less is known regarding sulfatide toxicity in astrocytes. Poly (ADP-ribose) polymerase (PARP) inhibitors are anti-cancer therapeutics that have proven efficacy in preclinical models of many neurodegenerative and inflammatory diseases, but have never been tested for MLD. Here, we examined the toxic effect of sulfatides on human astrocytes and restoration of this cell damage by the marketed PARP-1 inhibitor, Olaparib. Cultured human astrocytes were treated with increasing concentrations of sulfatides (5–100 μM) with or without Olaparib (100 nM). Cell viability assays were used to ascertain whether sulfatide-induced toxicity was rescued by Olaparib. Immunofluorescence, calcium (Ca^2+^) imaging, ROS, and mitochondrial damage assays were also used to explore the effects of sulfatides and Olaparib. ELISAs were performed and chemotaxis of peripheral blood immune cells was measured to examine the effects of Olaparib on sulfatide-induced inflammation in human astrocytes. Here, we established a concentration-dependent (EC_50_∼20 μM at 24 h) model of sulfatide-induced astrocyte toxicity. Our data demonstrate that sulfatide-induced astrocyte toxicity involves (i) PARP-1 activation, (ii) pro-inflammatory cytokine release, and (iii) enhanced chemoattraction of peripheral blood immune cells. Moreover, these sulfatide-induced effects were attenuated by Olaparib (IC_50_∼100 nM). In addition, sulfatide caused impairments of ROS production, mitochondrial stress, and Ca^2+^ signaling in human astrocytes, that were indicative of metabolic alterations and that were also alleviated by Olaparib (100 nM) treatment. Our data support the hypothesis that sulfatides can drive astrocyte cell death and demonstrate that Olaparib can dampen many facets of sulfatide-induced toxicity, including, mitochondrial stress, inflammatory responses, and communication between human astrocytes and peripheral blood immune cells. These data are suggestive of potential therapeutic utility of PARP inhibitors in the sphere of rare demyelinating diseases, and in particular MLD.

**Graphical Abstract:**

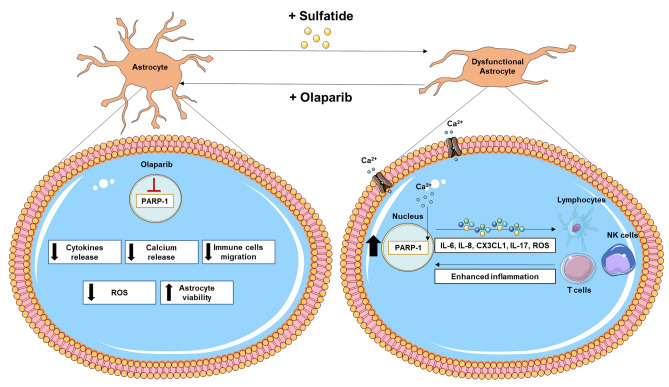

**Graphical abstract. Proposed mechanism of action of Olaparib in sulfatide-treated astrocytes.** Human astrocytes treated for 24 h with sulfatides increase PARP-1 expression and die. PARP-1 overexpression is modulated by Ca^2+^ release from the endoplasmic reticulum, thus enhancing intracellular Ca^2+^ concentration. PARP-1 inhibition with Olaparib reduces Ca^2+^ influx and cell death. Olaparib also decreases IL-6, IL-8, IL-17, and CX3CL1 release from sulfatide-stimulated astrocytes, suggesting that PARP-1 plays a role in dampening neuroinflammation in MLD. This is confirmed by the reduction of immune cell migration such as lymphocytes, NK cells, and T cells towards sulfatide-treated astrocytes. Moreover, mitochondrial stress and ROS production induced by sulfatides are rescued by PARP-1 inhibition. Future studies will focus on the signaling cascades triggered by PARP-1-mediated currents in reactive astrocytes and Olaparib as a potential therapeutic target for MLD.

**Supplementary Information:**

The online version contains supplementary material available at 10.1007/s11481-023-10092-9.

## Background

Metachromatic leukodystrophy (MLD) is a demyelinating, autosomal recessive genetic leukodystrophy with an estimated birth prevalence of approximately 1–2/100,000, and an incidence of 1/40,000 births [15]. MLD is a progressive disease for which there is currently no curative therapy. This disease is associated with the loss of muscle and cognitive function, as well as progressive loss of vision (Gieselmann 2003). Lifespan often depends on the age of diagnosis. In the late infantile form of this disease, death typically occurs within 5–6 years, while juvenile and adult forms may facilitate survival until early adulthood or more (Gieselmann 2003). The disease is caused by mutations in the arylsulfatase A gene (ARSA) and, consequently, a deficient activity of the arylsulfatase A lysosomal enzyme (Biffi et al. 2008). The loss of function in ARSA enzyme activity leads to the accumulation of galactosylceramide-3-O-sulfate (sulfatide) in the central and peripheral nervous systems (Rosenberg et al. 2016). Sulfatide accumulates mainly in oligodendrocytes, resulting in the dysfunction and destruction of the neuronal myelin sheaths, as well as the inhibition of oligodendrocyte differentiation from precursor cells, thereby preventing remyelination (Rosenberg et al. 2016).

Several hypotheses have been formulated for the involvement of sulfatides in MLD neurodegeneration and their contribution to MLD features, such as, disruption of the blood–brain barrier (BBB), dysfunctional transcription, translation, and expression of essential proteins for myelin, and neuroinflammation (Dali et al. 2015). The reported studies investigating sulfatide-induced cell damage have focused mainly on the role of the ARSA mutation in oligodendrocytes (Takahashi and Suzuki 2012). Investigating the role of astrocytes in MLD may provide additional opportunities in understanding this disease as well as the development of novel therapies. Astrocytes play an essential role in supporting myelination by producing the major fraction of brain cholesterol, where an absence in astrocyte cholesterol production results in hypomyelination (Camargo et al. 2017). In addition, astrocytic endfeet that express the water channel aquaporin 4 (AQP4) and the Kir4.1 K + channel, are one of the major regulators of BBB integrity (Abbott et al. 2006). Astrocytes also participate in innate immune responses and neuroinflammation, which is a key driver of MLD disease progression, with elevated levels of CCL2, interleukin-1 receptor antagonist (IL-1Ra), interleukin 8 (IL-8), and CCL4 in the cerebral spinal fluid of patients with MLD (Thibert et al. 2016). Furthermore, excessive glial cell proliferation and microglia hyperactivation are observed in a mouse model of MLD (Eichler and Van Haren 2007). Thus, it is conceivable to speculate that astrocytes are affected by the accumulation of sulfatides and contribute strongly to axonal degeneration and chronic inflammation in MLD.

Poly (ADP-ribose) polymerase (PARP) inhibitors are anti-cancer therapeutics that have proven efficacy in preclinical models of many neurodegenerative and inflammatory diseases such as stroke, Parkinson’s disease, Alzheimer’s disease, multiple sclerosis, diabetes, and myocardial infarction (Mekhaeil et al. 2022; Gariani et al. 2017; Sachdev et al. 2019; Sriram et al. 2014). PARP-1 is the most abundant and well-characterized member of the PARP nuclear enzyme superfamily that catalyses the transfer of ADP-ribose units from nicotinamide adenine dinucleotide (NAD+) to a broad panel of acceptor proteins such as histones and transcription factors. PARP-1 is involved in a wide range of cellular processes including DNA repair, death signaling, transcriptional regulation, mitochondrial function, and inflammation. PARP-1-mediated cell death and inflammation have been implicated in the pathogenesis of several CNS diseases (Kauppinen et al. 2005). PARP-1 plays a role in disease via co-activation of nuclear factor kappa-light-chain-enhancer of activated B cells (NF-κB), which induces the transcription of genes encoding proteins such as inducible nitric oxide synthase (iNOS), tumor necrosis factor α (TNF-α), cell adhesion molecules (e.g., intercellular adhesion molecule-1 (ICAM-1), vascular adhesion molecule-1 (VCAM-1)), interleukin-1 (IL-1), and interleukin-6 (IL-6) (Swindall et al. 2013; Schreiber et al. 2006). Together these molecules enhance inflammation, which in turn augments the expression of reactive oxygen species (ROS) and increases genomic instability as well as the sensitivity of surrounding cells to oxidation (Swindall et al. 2013). In addition, NF-κB induces further PARP-1 activation, thus creating a chronic oxidative loop (Ke et al. 2019). PARP-1 overactivation also causes excessive poly (ADP-ribose) (PAR) synthesis, NAD+ and ATP depletion, and ultimately, cell death (Schreiber et al. 2006).

Several FDA-approved PARP inhibitors, particularly Olaparib and Veliparib, have shown efficacy in reducing oxidative stress, decreasing PAR synthesis, preventing NAD+ depletion and cell death, decreasing NF-κB activation, and reducing expression of adhesion molecules and lymphocyte infiltration in neurological disease (Scott et al. 2004). Considering the evidence of augmented DNA fragmentation, oxidative stress, inflammation, as well as mitochondrial dysfunction, and BBB disruption in MLD pathology, the focus of this study was to; (i) uncover the effects of sulfatide accumulation in human astrocytes; (ii) determine whether Olaparib-mediated PARP inhibition could exert a protective effect against sulfatide-induced astrocyte dysfunction; and (iii) elucidate the mechanisms underpinning sulfatide-induced astrocyte toxicity.

## Materials and Methods

### Pharmacological Compounds

Sulfatide (Sigma; 383906-24-9) comprises the major glycolipid components of myelin and was prepared as a 10 mM stock solution dissolved in 90% dimethyl sulfoxide (DMSO, Sigma; D8418). Olaparib (Bioscience; AZD2281) is a PARP-1 inhibitor (IC50 = 13 nM) (Steffen et al. 2013) with partial binding affinity for PARP-2 that was prepared as a 20 mM stock solution in 90% DMSO.

### Human Astrocyte Cell Culture

Human astrocytes from fetal brains were purchased from ScienCell Research Laboratory, USA (1800, Lot Nos. 9063 and 11065), as per the ethics outlined by the supplier and as we have described previously (Elain et al. 2014; Rutkowska et al. 2015). Human astrocytes were cultured at 37 °C and 5% CO2 in a humidified incubator and grown in standard DMEM/F12 media (Fisher; 10770245) supplemented with 1% astrocyte growth supplement (ScienCell; 1852), 10% fetal bovine serum (FBS, Sigma; F7524) and 1% penicillin/streptomycin (pen/strep, Sigma; P4333) in T75 culture flasks (Corning) unless otherwise indicated in the figure legends. Human astrocytes were grown for 14 days until 90% confluent and then seeded into 24-well or 96-well plates until 80% confluent.

### MTT Assay

Astrocytes were plated in 96-well plates and cultured for 24 h until 80% confluent. The cells were starved with serum-free media (DMEM/F12) for 4 h, treated with or without compounds, after which the media was removed and replaced by 100 μL of fresh, pre-warmed media supplemented with 10 μL of 12 mM MTT (Invitrogen, M6494). The cells were incubated with MTT for 4 h at 37 °C. After 4 h of incubation, 75 μL of media was removed from each well and 50 μL of DMSO was added. The cells were incubated for 10 min at 37 °C. The plate was shaken and the absorbance read at 540 nm.

### Immunocytochemistry

Human astrocytes were plated in 24-well plates (Corning) and cultured for 24 h until 80% confluent. After pharmacological treatment, cells were washed in PBS and fixed in 4% paraformaldehyde (PFA) for 5 min. Cells were washed for 3 × 7 min in PBS and then permeabilized by incubation with 0.1% Triton-X-100 in PBS for 5 min at room temperature. Another 3 × 7 min PBS washes were performed, and non-reactive sites were blocked by adding 1% bovine serum albumin (Sigma) in PBS for 1 h. The cells were then incubated in primary antibody mouse monoclonal anti-PARP-1 (Santa Cruz; sc-8007; 1/300 dilution), mouse monoclonal anti-pADPr (Santa Cruz; sc-56198; 1/500 dilution), or mouse monoclonal anti-vimentin (Santa Cruz, sc-373717; 1/500 dilution) overnight at 4 °C. The primary antibody was removed, and the cells were washed for 3 × 7 min PBS after which the secondary fluorescent antibody goat anti-mouse Alexa fluor 488 (Invitrogen, A11001; 1/1000 dilution), was applied for 1 h at room temperature. After washing three times in PBS, the cells were then incubated with Hoechst nuclear stain (diluted 1:10,000 in PBS) (Invitrogen; H21486) for 10 min and washed twice again. The coverslips were then mounted on glass slides in Vectashield (Vector Labs) and the edges were sealed with varnish. Samples were stored at 4 °C in the dark until imaged. Images were captured with a confocal microscope (Leica SP8) in 1024 × 1024 resolution at 200 frames per second (fps).

### Microscopy and Image Analysis

Immunofluorescence images were captured at ×20 or ×40 magnification using a Leica SP8 confocal microscope. The images were exported as 8-bit.tif files for analysis using the software package FIJI (ImageJ, NIH, version 2.0.0). To analyze astrocyte morphology, FIJI’s region of interest (ROI) manager software plugin was used to identify individual cells in each image, enabling the calculation of both fluorescence intensity and cell area. For PARP-1 and PAR, the fluorescence images were processed as follows: ROIs of cellular nuclei automatically generated by ImageJ using the Hoechst field were utilized to quantify the average gray value of the PARP-1 or PAR field.

### Western Blot

Human astrocyte protein samples were obtained by scraping the cell monolayer in RIPA buffer. Samples were run in a 10% SDS-PAGE electrophoresis gel and transferred to PVDF membranes (Millipore, IPVH00010) by wet transfer (buffer composition: 2.5 mM Tris, 19.2 mM Glycine and 5% methanol, pH 8.3) at constant 70 V for 3 h on ice. Membranes were then blocked in PBS containing 5% BSA and 0.05% Tween-20 for 2 h at room temperature and incubated with a primary antibody that binds PARP-1 (SantaCruz, sc-8007; dilution 1/300 in blocking buffer) for 18 h at 4 °C. After several PBS and PBS-Tween 0.05% washes, membranes were incubated with goat anti-mouse secondary antibody (Sigma, A8924; RRID: AB 258426; dilution 1/5,000 in blocking buffer) for 2 h at room temperature. Several washes were then performed and PVDF membranes were developed using a chemiluminescent HRP substrate (Millipore, WBKLS0500).

### JC-1 Assay

Changes in mitochondrial potential were measured using a commercially available tetraethylbenzimidazolylcarbocyanine iodide (JC-1) assay kit (Abcam, ab113850) following the manufacturer's instructions. Briefly, astrocytes were seeded in a 24-well plate at a density of 30,000 cells/well and treated with sulfatides (5 µM, 10 µM, 20 µM, 50 µM, and 100 µM) and Olaparib (100 nM). Thirty minutes before the end of each treatment, 1 μM JC-1 dye was added to each well. After 30 min, the wells were washed twice with dilution buffer. The excitation wavelength was set at 535 ± 17.5 nm and the emission at 590 ± 17.5 nm. Aggregate emission was measured in a BioTek synergy HT microplate reader (BioTek, VT).

### Reactive Oxygen Species Assay

Human astrocytes were seeded in a 96-well plate at a density of 20,000 cells/well for 24 h and treated with sulfatides (5 µM, 10 µM, 20 µM, 50 µM, and 100 µM) and Olaparib (100 nM). Cellular ROS was measured using a detection assay kit (Merck; D6883). In brief, 2′,7′-dichlorofluorescein diacetate (DCFDA), a fluorogenic dye that measures hydroxyl, peroxyl, and other ROS activity within the cell. After diffusion into the cells, DCFDA was deacetylated by cellular esterases to a non-fluorescent compound, which was later oxidized by ROS into 2′,7′-dichlorofluorescin (DCF), a highly fluorescent compound. Fluorescence from the DCF was detected by a fluorescence microplate reader with maximum excitation and emission spectra of 495 nm and 529 nm, respectively.

### Live-Cell Calcium Imaging

Astrocytes were seeded in a 24 well-plate and grown for 24 h in supplemented DMEM/FBS until 80% confluent. Cells were then serum-starved for 4 h prior to treatment with sulfatides (5 µM, 10 µM, 20 µM, 50 µM, and 100 µM) with or without 100 nM Olaparib for 24 h. After treatment, cells were incubated with 3 μM Cal-520AM dye (Abcam, ab171868) in Hank's balanced salt solution (HBSS; no phenol red) supplemented with 10 mM glucose and 25 mM HEPES for 90 min at 37 °C, followed by 30 min at room temperature. Cells were protected from bright light at all times. Cells were then incubated in HBSS minus Ca^2+^ and Mg^2+^ (HBSS−/−). Time-lapse Ca^2+^ imaging was performed at a rate of 0.99 frames per second. Following a 20 s baseline, cells were stimulated with 300 μM ATP for 240 s. Intracellular Ca^2+^ imaging experiments were performed with HBSS minus Ca^2+^ and Mg^2+^ (HBSS−/−). Images were acquired using a motorised epifluorescent microscope and a ×20 magnification objective.

### Enzyme-Linked Immunosorbent Assay (ELISA)

All cells were starved in serum-free media for 4 h before treatments. Human astrocytes were treated with sulfatides with or without Olaparib for 24 h. Media were then collected, and frozen at −80 °C. Soluble fractalkine (sCX3CL1), interleukin 6 (IL-6), interleukin 8 (IL-8), and interleukin 17 (IL-17) levels in astrocyte conditioned media were detected with human CX3CL1 ELISA kit according to the manufacturer’s instructions (R&D Systems; DY365), human IL-6 kit (R&D Systems; DY206), human IL-8 kit (R&D Systems; DY208), and human IL-17 (R&D Systems; DY317). Briefly, 96-well ELISA plates (Thermo Scientific; 95029780) were coated overnight at 4 °C with capture antibodies diluted in Dulbecco's PBS (dPBS, Sigma; 14190–094). The plates were washed three times with wash buffer (0.05% Tween 20 (Sigma; P7949), 10X PBS, pH 7.4) and then blocked for 2 h at room temperature with the appropriate reagent diluent. The plates were then washed three times with wash buffer, and any remaining buffer was removed from the wells by aspiration. A standard curve was prepared using serial dilutions of the recombinant protein diluted in the appropriate reagent diluents. The samples and standards were then incubated in the antibody-coated ELISA plate for 2 h at room temperature. The plate was then washed three times with wash buffer, and a detection antibody (diluted in reagent diluent) was added to each well for 2 h. Following three more washes, streptavidin-HRP diluted in reagent diluents was added to each well and incubated for 20 min at room temperature, protected from light. After an additional three washes, the wells were incubated with substrate solution (R&D systems; DY999) for 15 min at room temperature protected from light. The color reaction was stopped with the addition of 1 M H2SO4, and absorbance was read immediately using a plate reader at 450 nm (Labsystem Multiskan). The standard curve was calculated by plotting the standards against the absorbance values, and the cytokine levels were measured in pg/ml or ng/mL.

### Chemotaxis Assay

Peripheral blood samples were taken from 6 healthy donors. Peripheral blood mononuclear cells (PBMC) were isolated from the peripheral blood by density gradient centrifugation and resuspended in RPMI supplemented with 10% fetal bovine serum (FBS, Sigma; F7524) and 1% penicillin/streptomycin (pen/strep, Sigma; P4333) for 24 h. Conditioned media from astrocyte cell cultures (treated with or without sulfatides and with or without Olaparib) were added to the lower chamber of a 5 µm pore Transwell filter system (Corning Inc, Corning, NY, USA). Serum-free DMEM was used as a negative control and DMEM supplemented with 20% FBS was used as a positive control for chemotaxis. PBMC were subsequently added at a density of 0.2 × 10^6^ cells/100 µL RPMI to the upper chamber of the transwell system. This system was incubated for 2 h at 37 °C, with 5% CO_2_. Migrated cells were collected from the lower chamber and stained for flow cytometric analysis with CD56-FITC-Viobright (Miltenyi Biotec) and CD3-APC-Cy7 (Biolegend). CountBright beads (ThermoFisher, Waltham, MA, United States) were used to enumerate the migrated lymphocytes, CD56^+^CD3^−^ NK (Natural Killer) cells, and CD56^−^CD3^+^ T cells. Cells were acquired using the CANTO II flow cytometer (BD Biosciences) and analyzed using FlowJo v10 software (Tree Star).

### Statistical Analysis

All data were analyzed using GraphPad Prism 5 (GraphPad Software). One-way and Two-way analysis of variance (ANOVA) and multiple comparisons post hoc tests were used to assess significant differences between the values obtained for Control, sulfatides- and drug-treated astrocytes. The differences were considered significant if p < 0.05 and all values were expressed as the mean “standard error of the mean” (SEM) or standard deviation (SD). Where indicated, ‘n’ stands for the number of separate experiments carried out. Two or three technical replicates were performed for each condition/drug treatment within each separate experiment. For human astrocytes, a separate experiment is counted as experiments done with cells from a different passage. No blinding of the researcher was performed for the in vitro component of this study.

## Results

### Sulfatides Induce Human Astrocyte Cell Death

**Fig. 1:**
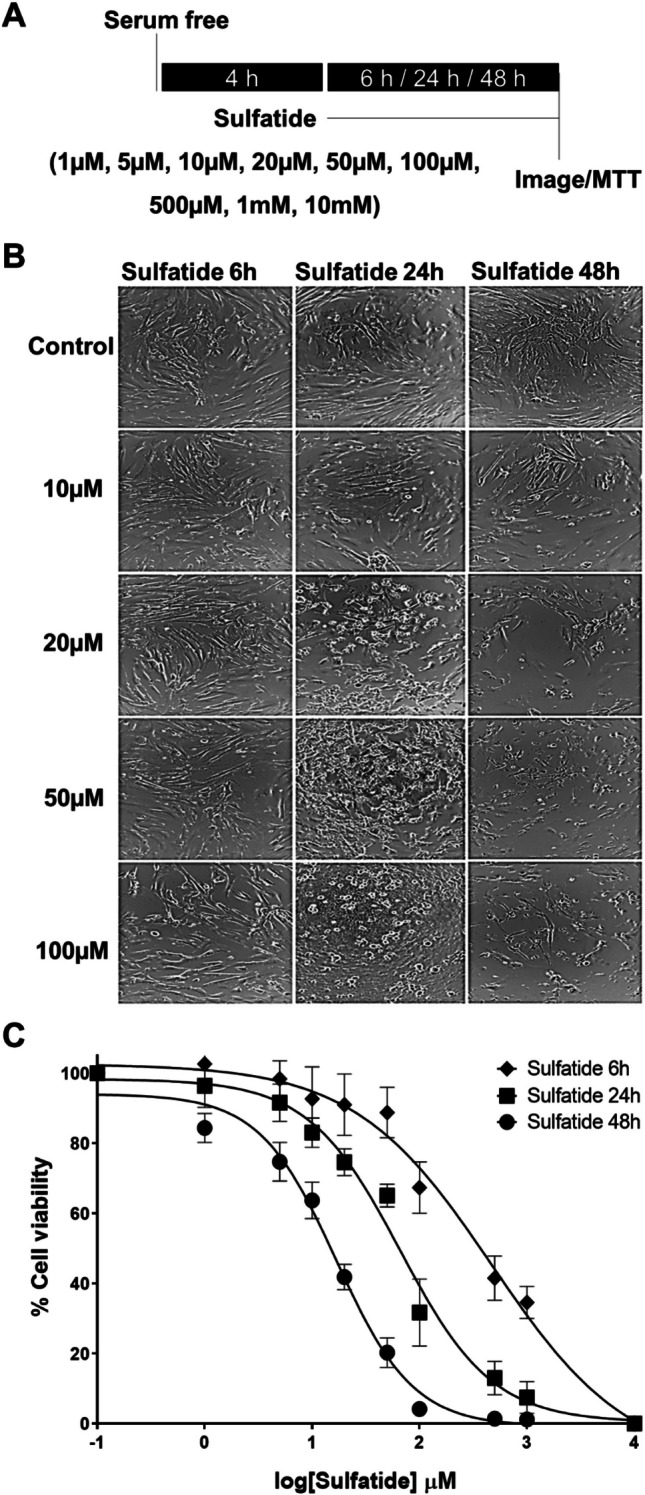
**Sulfatides induce human astrocyte cell death. A** Diagram of experimental timeline depicting astrocytes were incubated in serum-free media for 4 h and treated with sulfatides at concentrations indicated for 6 h or 24 h or 48 h. **B** Representative images of human astrocytes treated with 10 µM, 20 µM, 50 µM, and 100 µM sulfatides for either 6 h, 24 h, or 48 h. Cells were imaged under light microscopy. **C** Dose–response curves showing that sulfatides decreased human astrocyte viability in a concentration-dependent (5 µM, 10 µM, 20 µM, 50 µM, and 100 µM) and time-dependent (6 h (black diamond), 24 h (black square), 48 h (black circle)) manner (n = 5–10). Cell viability was quantified by measuring NADP(H) activity using an MTT assay (Vybrant® MTT Cell Proliferation Assay Kit, Life technologies). All values are given as a percentage normalized to the control group. Statistical analysis was performed using a log dose–response test with GraphPad Prism 5. Graphical data are presented as mean ± SD

Sulfatide-mediated cell toxicity of astrocytes is less well studied in comparison to the effects of this lipid on oligodendrocytes. Therefore, the effects of sulfatides on human astrocyte survival were first investigated. Cultured human astrocytes were serum-starved for 4 h and then treated with sulfatides at the timepoints and concentrations indicated (Fig. 1A). Sulfatides reduced human astrocyte numbers in a concentration- and time-dependent manner: sulfatides treatment for 6 h modestly reduced astrocyte survival (20 µM, 92.6%; 50 µM, 88.3%; and 100 µM, 68.1%, compared with control; mean ± SEM). Treatment with sulfatides for 24 h and 48 h significantly reduced astrocyte cell survival (for 24 h; 20 µM, 74.2%; 50 µM, 65.8%; and 100 µM, 48.2%, compared with control; mean ± SEM) (for 48 h; 20 µM, 42.4%; 50 µM, 20.7%; and 100 µM, 4.7%, compared with control; mean ± SEM) (Fig. 1B and C). These data suggest that sulfatides induce astrocyte dysfunction and may contribute to demyelination in the associated disease.

### Olaparib Reduces Sulfatide-Induced Toxicity in Human Astrocytes

**Fig. 2:**
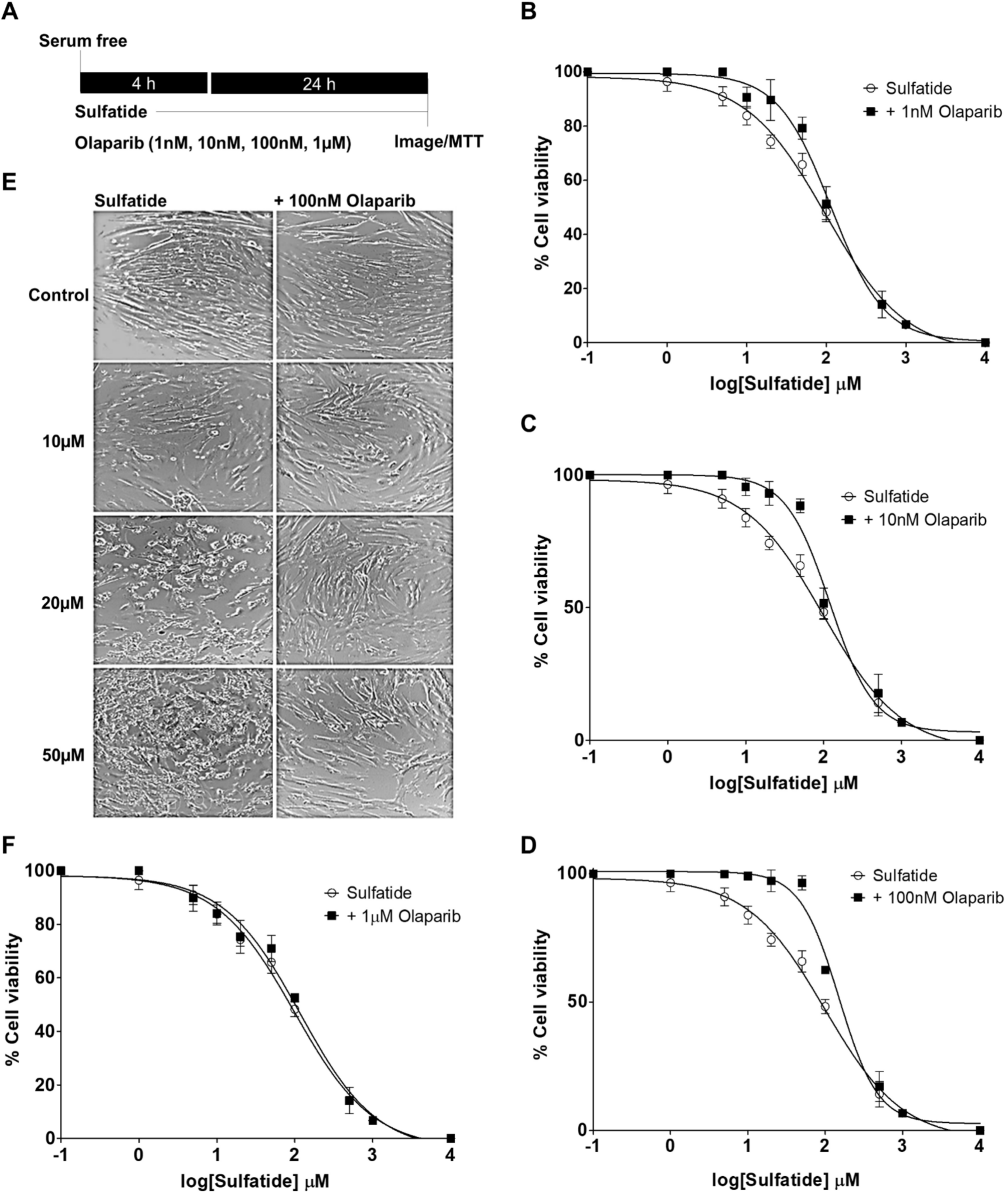
**Olaparib reduces sulfatide-induced human astrocyte cell death. A** Diagram of experimental timeline and treatments. Human astrocytes were treated with 5 µM, 10 µM, 20 µM, 50 µM, and 100 µM sulfatides for 24 h with or without 100 nM Olaparib. **B** A dose–response curve showing changes in astrocyte viability after treatment with sulfatides (5 µM, 10 µM, 20 µM, 50 µM, and 100 µM, clear circle) with or without Olaparib treatment (1 nM, black square) for 24 h (n = 5). **C** A dose–response curve showing changes in astrocyte viability after treatment with sulfatides (5 µM, 10 µM, 20 µM, 50 µM, and 100 µM, clear circle) with or without Olaparib treatment (10 nM, black square) for 24 h (n = 5). **D** A dose–response curve showing changes in astrocyte viability after treatment with sulfatides (5 µM, 10 µM, 20 µM, 50 µM, and 100 µM, clear circle) with or without Olaparib treatment (100 nM, black square) for 24 h (n = 10). **E** Representative images of astrocytes treated with sulfatides (20 µM, 50 µM, and 100 µM) with or without Olaparib (100 nM) for 24 h. Cells were imaged under light microscopy. **F** A dose–response curve showing changes in astrocyte viability after treatment with sulfatides (5 µM, 10 µM, 20 µM, 50 µM, and 100 µM, clear circle) with or without Olaparib treatment (1 µM, black square) for 24 h (n = 5). Cell viability was quantified by measuring NADP(H) activity using an MTT assay (Vybrant® MTT Cell Proliferation Assay Kit, Life Technologies). Statistical analysis was performed using a log dose–response test with GraphPad Prism 5. Graphical data are presented as mean ± SD

Given the data above demonstrating that sulfatides induce cell toxicity in human astrocytes in a time-dependent manner, all further studies were carried out investigating the effects of sulfatide treatment at 24 h. Next, the protective effects of Olaparib on sulfatide-induced toxicity in human astrocytes were examined. Olaparib is a poly (ADP-ribose) polymerase (PARP) inhibitor, marketed as an anticancer therapeutic (Bochum et al. 2018). Human astrocytes were treated with sulfatides (1 µM, 5 µM, 10 µM, 20 µM, 50 µM, 100 µM, 500 µM, 1 mM, and 10 mM) and Olaparib (1 nM, 10 nM, 100 nM, and 1 µM) at the time points indicated (Fig. 2A). Olaparib demonstrated a protective effect at low doses (1 nM, 10 nM, and 100 nM) on sulfatide-induced astrocyte cell death (1 nM Olaparib: 10 µM, 83.8% vs 90.6%; 20 µM, 74.2% vs 89.6%; 50 µM, 65.8% vs 79.2%;100 µM, 48.2% vs 51.2%, mean ± SEM) (Fig. 2B), (10 nM Olaparib: 10 µM, 83.8% vs 95.4%; 20 µM, 74.2% vs 93.0%; 50 µM, 65.8% vs 88.3%;100 µM, 48.2% vs 51.6%, mean ± SEM) (Fig. 2C), (100 nM Olaparib: 10 µM, 83.8% vs 99.0%; 20 µM, 74.2% vs 97.1%; 50 µM, 65.8% vs 96.3%; 100 µM, 48.2% vs 59.1%, mean ± SEM) (Fig. 2D and E), (1 µM Olaparib: 10 µM, 83.8% vs 84.4%; 20 µM, 74.2% vs 75.3%; 50 µM, 65.8% vs 70.9%;100 µM, 48.2% vs 52.7%, mean ± SEM) (Fig. 2F). These results demonstrate Olaparib's efficacy in counteracting sulfatide-induced cytotoxicity in human astrocytes and further underline that the efficacy is most pronounced at the 100 nM dosage of Olaparib. Therefore, this concentration of Olaparib was selected for subsequent experiments.

### Olaparib Decreases Sulfatide-Induced PARP-1 Activation in Human Astrocytes

**Fig. 3:**
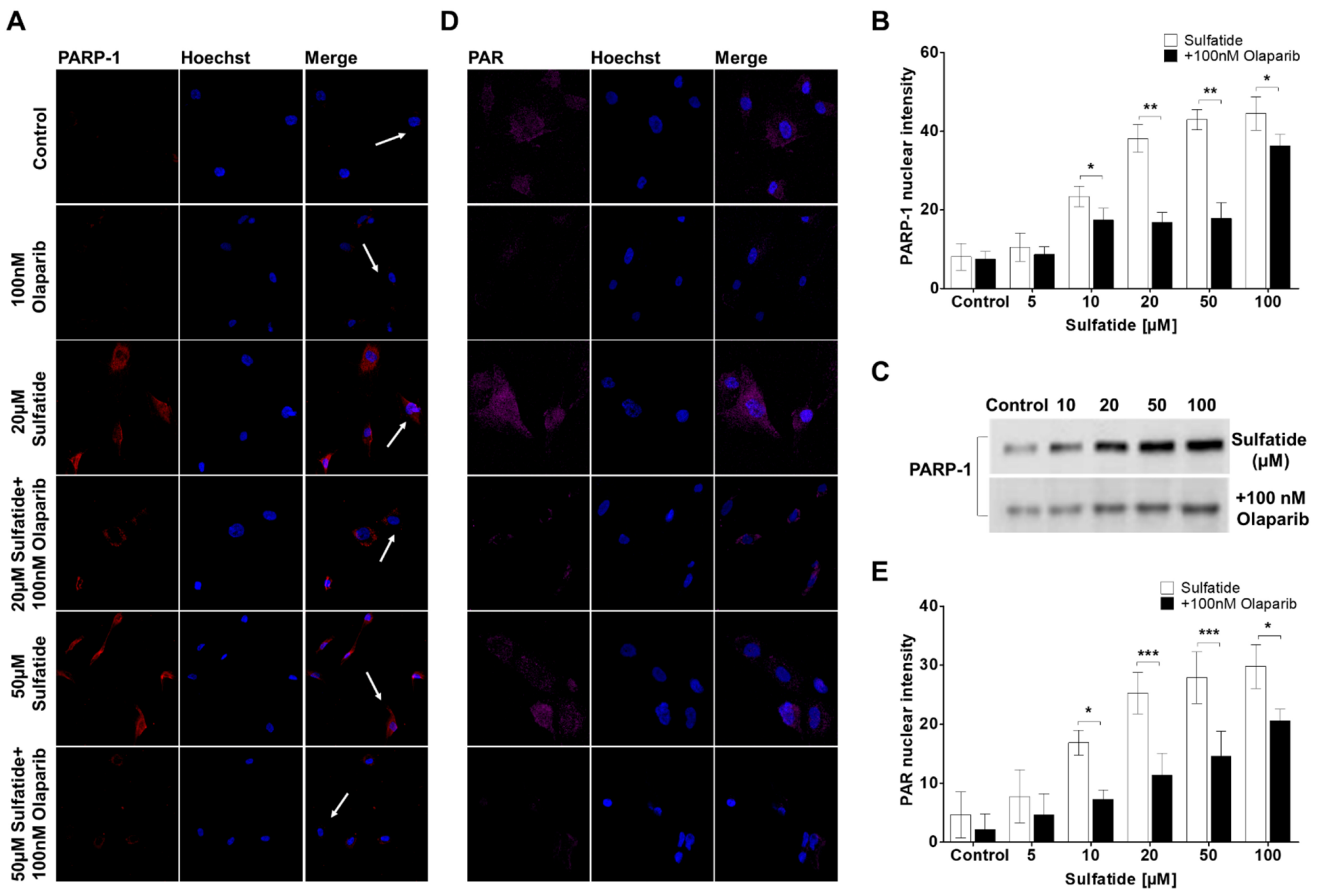
**Olaparib decreases sulfatide-induced PARP-1 activation and expression in human astrocytes. A** Representative confocal images displaying PARP-1 (red) and Hoechst (blue) immunostaining under treatment conditions indicated. Confocal images were captured at ×40 magnification. **B** Treatment with Olaparib (100 nM) reduced sulfatide-induced PARP-1 translocation to nuclei (5 µM, 10 µM, 20 µM, 50 µM, and 100 µM). A number of 15 images were analyzed per condition. Semi-quantitative analysis of PARP-1-associated fluorescence in the nuclei of astrocytes. **C** To confirm the effects of sulfatides and Olaparib on PARP-1, a western blot was conducted and showed that PARP-1 protein levels were increased after sulfatide treatment in a concentration-dependent manner. Olaparib reduced sulfatide-induced PARP-1 expression. **D** Representative confocal images displaying PAR (purple) and Hoechst (blue) immunostaining under treatment conditions indicated. Confocal images were captured at ×63 magnification. **E** Treatment with Olaparib (100 nM) reduced sulfatide-induced PAR expression in nuclei (5 µM, 10 µM, 20 µM, 50 µM, and 100 µM). A number of 15 images were analyzed per condition. Data are presented as mean ± SEM (n = 5), one-way ANOVA followed by Turkey’s post hoc test, *p < 0.05, **p < 0.01, ***p < 0.001

Under normal physiological conditions, PARP-1 activity is involved in a myriad of cellular processes that includes detection and repair of DNA damage, stress response, and chromatin maintenance (Bai 2015). In neurodegenerative disorders including Parkinson’s disease and multiple sclerosis, hyperactivation of PARP-1 results in neuronal death via accumulation of PAR polymers as well as excessive glial cell activation (Alano et al. 2010). Hence, the effects of sulfatides and Olaparib on PARP-1 activation in human astrocytes were investigated by measuring the changes in PARP-1 expression in the nucleus. Treatment of human astrocytes with sulfatides (5 μM; 10 μM; 20 μM; 50 μM; 100 μM) for 24 h induced PARP-1 expression in astrocyte nuclei in a concentration-dependent manner (Fig. 3A and Supplementary Fig. 1C). Importantly, these changes in PARP-1 fluorescence were reduced by treatment with Olaparib (100 nM) (Ctrl: 8.0 vs 7.4; 5 μM 10.4 vs 8.6; 10 μM 23.4 vs 17.3, *p < 0.05; 20 μM 38.1 vs 16.8, **p < 0.01; 50 μM 42.9 vs 17.8, **p < 0.01; 100 μM 44.4 vs 36.2, *p < 0.05) (Fig. 2B). Results obtained through immunofluorescence were confirmed by Western blot where a detectable increase in PARP-1 protein (~132 kDa band) was observed in astrocytes after 24-h sulfatides exposure, with a consequent decrease following Olaparib treatment (Fig. 3C). The assessment of PAR synthesis serves as a representative marker of PARP-1 activation. Accordingly, PAR expression was quantified within the nuclei of astrocytes treated with sulfatides (5 μM; 10 μM; 20 μM; 50 μM; 100 μM) in the presence or absence of Olaparib (100 nM) (Fig. 3D and Supplementary Fig. 1D). Sulfatides induced a concentration-dependent elevation in PAR expression, which was significantly ameliorated by Olaparib treatment (Ctrl: 4.6 vs 2.2; 5 μM 7.7 vs 4.6; 10 μM 16.8 vs 7.2, *p < 0.05; 20 μM 25.2 vs 11.3, ***p < 0.001; 50 μM 27.8 vs 14.5, ***p < 0.001; 100 μM 29.7 vs 20.5, *p < 0.05) (Fig. 3E). Overall, these results support the idea that sulfatide-induced toxicity is at least in part mediated by PARP-1 overactivation and that Olaparib attenuates these effects.

### Sulfatide-Induced Impairment in Human Astrocyte Morphology is Partially Reversed by Olaparib

**Fig. 4:**
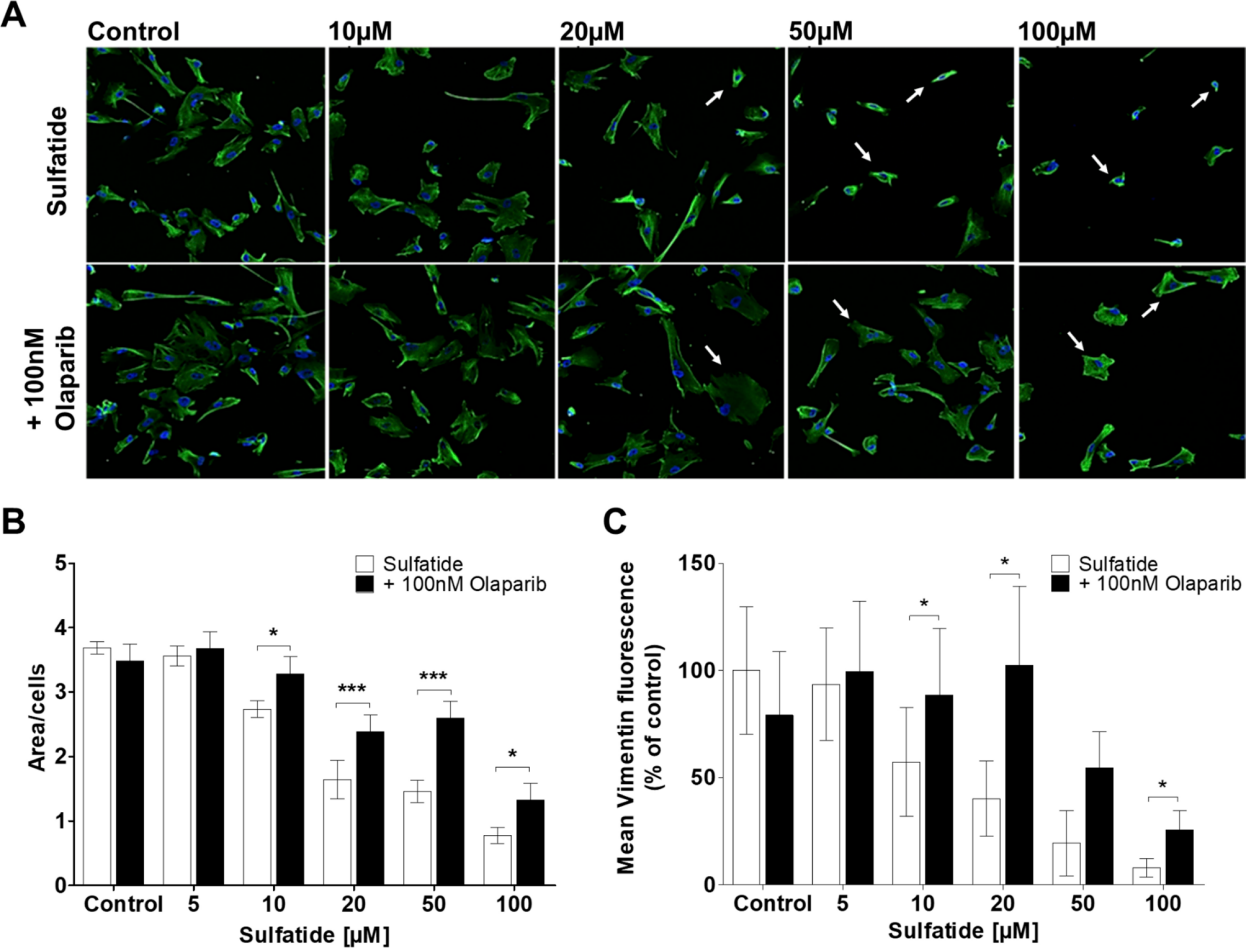
**Sulfatide-induced changes in vimentin in human astrocytes are partially reversed by Olaparib.** Human astrocytes were treated with sulfatides (5 µM, 10 µM, 20 µM, 50 µM, and 100 µM for 24 h) with or without the PARP inhibitor Olaparib (100 nM for 24 h). **A** Representative confocal images displaying Hoechst (blue) and vimentin (green) staining under treatment conditions indicated. Confocal images were captured at ×20 magnification showing that treatment with 100 nM Olaparib (bottom) attenuates astrocyte morphology impairment induced by sulfatides (5 µM, 10 µM, 20 µM, 50 µM, and 100 µM, top). A number of 15 images were analyzed per condition. **B** Bar graph illustrating changes in astrocytes area after the treatment with sulfatides (5 µM, 10 µM, 20 µM, 50 µM, and 100 µM) and with or without Olaparib (100 nM) for 24 h. **C** Bar graph illustrating changes in the intensity of vimentin fluorescence after the treatment with sulfatides (5 µM, 10 µM, 20 µM, 50 µM, and 100 µM) and with or without Olaparib (100 nM) for 24 h. Data are presented as mean ± SEM (n = 5), one-way ANOVA followed by Dunnett post hoc test, *p < 0.05, ***p < 0.001

To further examine the cytotoxic effects of sulfatides and the potential rescue by Olaparib, the effects of these treatments were investigated on changes in astrocyte morphology. Specifically, the protective effect of Olaparib on sulfatide-induced toxicity was examined by immunocytochemistry of type III intermediate filament astrocyte marker vimentin, which is involved in cytoskeleton formation in astrocytes. Treatment of human astrocytes with sulfatides (5 μM; 10 μM; 20 μM; 50 μM; 100 μM) for 24 h induced a reduction in vimentin expression in astrocytes, particularly in the cell processes, suggesting deregulation of the cellular cytoskeleton (Fig. 4A and Supplementary Fig. 1A and B). This change likely reflected the rounding of astrocytes before detachment induced by sulfatides. Importantly, these sulfatide-induced changes in vimentin localization were reduced by treatment with Olaparib (100 nM) (Ctrl: 3.6 vs 3.4; 10 µM, 2.7 vs 3.2, *p < 0.05; 20 µM, 1.6 vs 2.3, ***p < 0.001; 50 µM, 1.4 vs 2.5, ***p < 0.001, 100 µM, 0.7 vs 1.3, *p < 0.05) (Fig. 4B). The analysis of vimentin intensity fluorescence confirmed that Olaparib reverses sulfatide-induced astrocytes morphology impairment at 10 µM (7.46 vs 11.43, *p < 0.05), 20 µM (5.24 vs 13.36, *p < 0.05), and 100 µM (1.04 vs 4.20, *p < 0.05) (Fig. 4C). Overall, these results support the idea that altered cytoskeleton structure preceding sulfatide-induced cell death in human astrocytes is also attenuated by Olaparib.

### Olaparib Attenuates Sulfatide-Induced Oxidative Stress Damage and Mitochondrial Stress in Human Astrocytes

**Fig. 5:**
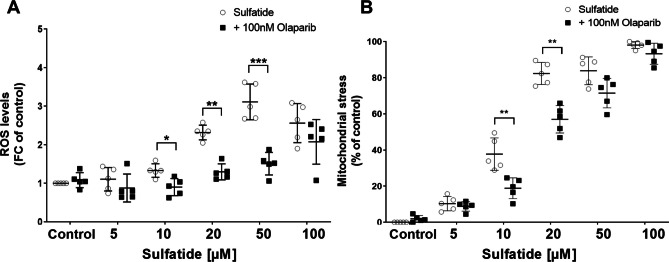
**Olaparib suppresses reactive oxygen species (ROS) generation caused by sulfatides in human astrocytes and reduces mitochondrial stress. A** Quantification of ROS levels was determined by 2’-7’ dichlorofluorescin diacetate (DCFH-DA) staining in human astrocytes treated with 5 µM, 10 µM, 20 µM, 50 µM, and 100 µM of sulfatides (clear circle) with or without 100 nM of Olaparib (black square). Data are presented as the mean absorbance levels (at 490 nm). **B** To confirm that PARP-1 activation had negligible effects on cellular stress, the JC-1 assay was performed to measure changes in the mitochondrial membrane potential of sulfatides (5 µM, 10 µM, 20 µM, 50 µM, and 100 µM) and Olaparib (100 nM). Data are presented as the mean absorbance levels (at 570 nm). The measures of fluorescence signal are reported in the graphs as fold change (FC) or percentage of each condition versus the control and presented as mean ± SD (n = 5). Statistical significance was determined by paired t-test, *p < 0.05, **p < 0.01, ***p < 0.001

Next, oxidative stress in human astrocytes was assessed using DCFH-DA. Following exposure to sulfatides (5 μM, 10 μM, 20 μM, 50 μM, 100 μM), the fluorescence intensity of DCFH-DA was elevated compared to the control group (Fig. 5A and Supplementary Fig. 1D). Treatment with 100 nM Olaparib significantly attenuated sulfatide-induced reactive oxygen species (ROS) levels (Ctrl: 1.0 vs 1.08; 5 μM 1.10 vs 0.87; 10 μM 1.32 vs 0.90, *p < 0.05; 20 μM 2.31 vs 1.29, **p < 0.01; 50 μM 3.11 vs 1.50, ***p < 0.001; 100 μM 3.16 vs 2.27) (Fig. 5A). Increasing evidence now suggests the involvement of PARP in alterations of electron transport and loss of mitochondrial membrane potential (ΔΨm) (Wang et al. 2018). Here, the ΔΨm was measured using the membrane-permeant dye tetraethylbenzimidazolylcarbocyanine iodide (JC-1), which exhibits potential-dependent accumulation in mitochondria. An increase in JC‐1 monomers indicates a loss of mitochondrial membrane potential. Cultured human astrocytes were serum-starved and treated with sulfatides (5 μM, 10 μM, 20 μM, 50 μM, 100 μM) with or without Olaparib (100 nM). Cells were then loaded with 1 μM JC-1 and after 30 min the emission spectra were measured. Sulfatides treatment of astrocytes caused loss of ΔΨm compared with control, which is indicative of increased mitochondrial stress (Fig. 5B and Supplementary Fig. 1E). Notably, treatment with Olaparib attenuated the sulfatide-induced increase of mitochondrial stress (Ctrl: 0.0 vs 1.29; 5 μM 11.16 vs 6.76; 10 μM 35.87 vs 21.01, **p < 0.01; 20 μM 82.27 vs 58.23, **p < 0.01; 50 μM 83.44 vs 73.84; 100 μM 97.64 vs 76.01) (Fig. 5B). These data further demonstrate astrocyte cell damage in the presence of sulfatides and support the involvement of PARP-1 in modulating oxidative stress and mitochondrial dysfunction.

### Olaparib Reduces Calcium Influx in Sulfatide-Stimulated Astrocytes

**Fig. 6:**
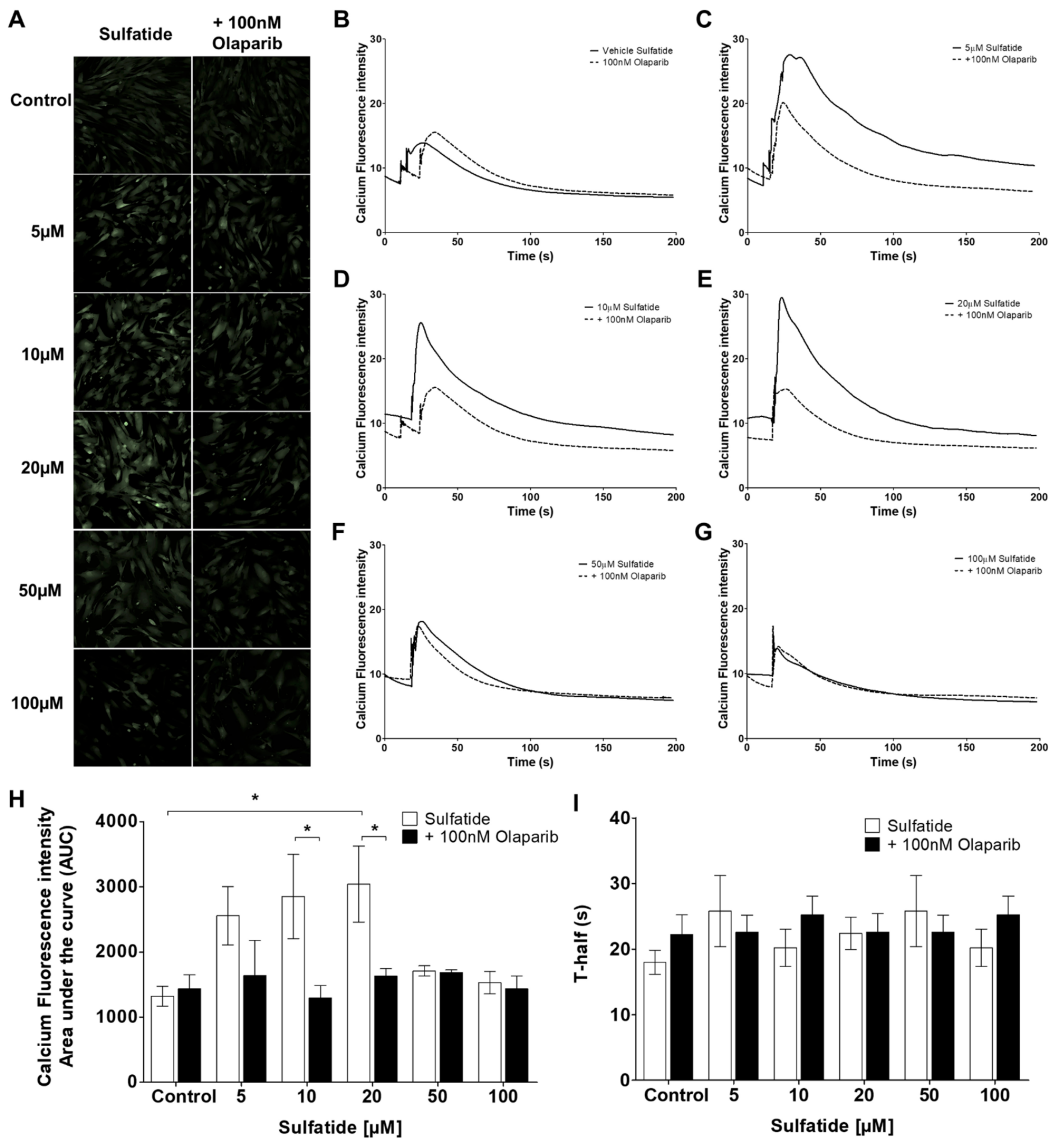
**Olaparib decreases** Ca^2+^ **changes caused by sulfatides in human astrocytes.** Human astrocytes were treated with 5 µM, 10 µM, 20 µM, 50 µM, and 100 µM sulfatides for 24 h and with or without 100 nM Olaparib and loaded with 3 µM Cal-520AM calcium indicator for 2 h in Hank's balanced salt solution (HBSS) without calcium (Ca^2+^) and magnesium (Mg^2+^). Immediately prior to Ca^2+^ imaging experiments, astrocytes were transferred to a fresh DMEM medium. Time-lapse Ca^2+^ imaging experiments were performed at a rate of 0.99 frames per second over a 3 min 20 s period, which included 20 s baseline, and 3 min of exposure to 300 μM ATP. **A** Representative confocal images (×20 magnification) of Ca^2+^ influx in response to 300 µM ATP in control and reactive astrocytes pre-treated with sulfatides (5 µM, 10 µM, 20 µM, 50 µM, and 100 µM) and with or without 100 nM Olaparib for 24 h. **B-G** Representative traces showing changes in cytosolic Ca^2+^ levels in controls and sulfatide-stimulated astrocytes (continuous line) with or without 100 nM Olaparib (broken line). There was a 30 s baseline, at which point astrocytes were stimulated with ATP for a further 3 min. **H-I** Olaparib (black square) caused a larger decrease of both the peak amplitude and AUC in 10 µM and 20 µM sulfatide-treated astrocytes (clear circle) but didn’t change the time half. Data for the total 3.20 min’ treatment period are presented as mean ± SEM (n = 5) and were analyzed using a two-way ANOVA followed by Bonferroni post-hoc test, *p < 0.05

Activation of PARP-1 has been observed to facilitate the expression of Ca^2+^ permeable channels and to alter mitochondrial Ca^2+^ homeostasis in ischemic or traumatic brain injury, and in a NMDA toxicity model in rat primary cortical neurons (Vosler et al. 2009; Gerace et al. 2015). Our results above show that sulfatides increase PARP-1 expression in human astrocytes (Fig. 2), hence the effects of sulfatides in the presence or absence of Olaparib on activation, influx, and oscillations of Ca^2+^ in human astrocytes were investigated. Astrocyte cultures were pre-treated with sulfatides (5 μM, 10 μM, 20 μM, 50 μM, 100 μM) with or without Olaparib (100 nM) for 24 h and then loaded with Cal-520AM calcium dye. Time-lapse Ca^2+^ imaging was performed. After a 20 s baseline recording, cells were treated with 300 μM ATP and imaged for a further 3 min (to determine the proportions of astrocytes that display ATP-induced Ca^2+^ oscillations). Sulfatide-treated astrocytes displayed ATP-induced Ca^2+^ peaks that were larger in amplitude than those elicited by astrocytes cotreated with sulfatides and Olaparib (Fig. 6A-G). Measurement of the area under the curve (AUC) of 20 µM sulfatides group showed the largest AUC Ca^2+^ response to ATP perfusion compared to the control group (1321.6 vs 3042.8, *p < 0.05). Interestingly, Olaparib significantly reduced this sulfatide-induced AUC Ca^2+^ response to ATP perfusion (10 µM, 2851.6 vs 1293.2, *p < 0.05; 20 µM, 3042.8 vs 1630.8, *p < 0.05) (Fig. 6H). Sulfatides treatment did not affect the halftime of Ca^2+^ release (Fig. 6I). Taken together with the data above showing sulfatide-induced PARP-1 overexpression and that Olaparib reduces Ca^2+^ oscillations in response to ATP in sulfatide-stimulated astrocytes, we speculate a cross modulation between the PARP-1 expression and Ca^2+^ signaling, that is regulated in an opposing manner by sulfatides and Olaparib.

### Olaparib Inhibits Pro-Inflammatory Cytokine and Chemokine Release from Sulfatide-Treated Astrocytes

**Fig. 7:**
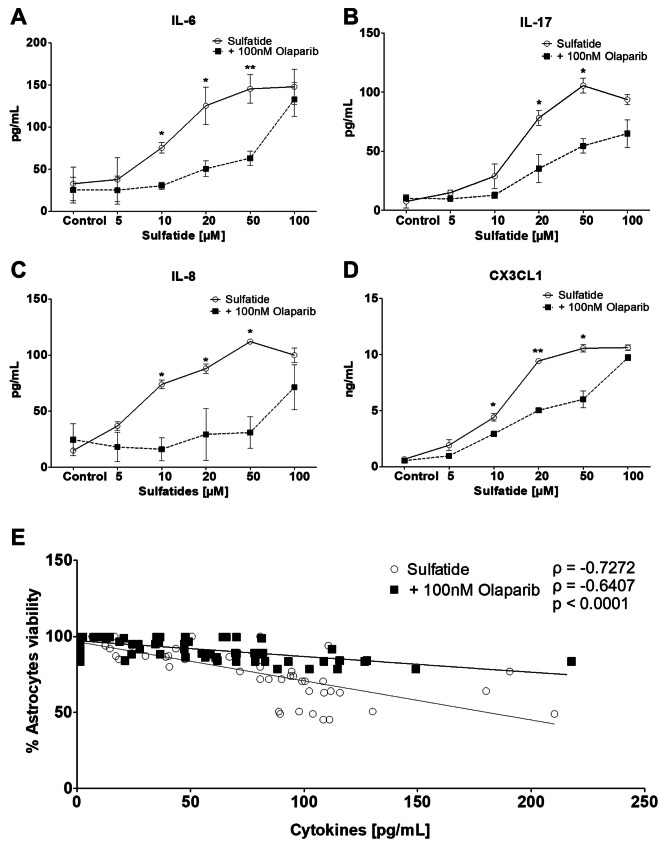
**PARP-1 modulates cytokine and chemokine release from human astrocytes.** Cytokine and chemokine release from control and sulfatide-stimulated astrocytes (clear circle) were measured after 24 h of Olaparib exposure (black square). Olaparib (100 nM) showed a significant decrease in **A** IL-6, **B** IL-17, **C** IL-8, and **D** CX3CL1 release into the cell culture medium, as measured by ELISA. **A** Olaparib causes a significant reduction in IL-6 secretion from astrocytes when compared to the sulfatides group, **B** Olaparib also caused a significant decrease in IL-17 secretion from sulfatide-stimulated astrocytes. **C** Olaparib reduces IL-8 secretion and **D** CX3CL1 from sulfatide-stimulated astrocytes. **E** Correlation between total cytokines level and astrocyte survival. The levels of the 4 cytokines were significantly positively correlated with cell death. Data from the ELISA assays are presented as mean ± SEM. IL-6, IL-17, IL-8, and CX3CL1 ELISA's were repeated n = 5 (using supernatants generated from MTT assay experiments). CX3CL1 values were multiplied by 1000 and divided by 100 to adjust Fig. E. Statistical analysis was performed using a paired t-test; *p < 0.05, **p < 0.01

MLD patients exhibit augmented levels of pro-inflammatory cytokines and chemokines including CCL2, IL-1Ra, IL-8, and CCL4 in the cerebral spinal fluid. PARP-1 has a key role in chronic inflammation in the context of many inflammatory-driven pathologies. Therefore, the effects of sulfatides treatment on the expression of pro-inflammatory cytokines and chemokines from human astrocytes were examined in the presence or absence of PARP-1 inhibition. The levels of pro-inflammatory cytokines IL-6, IL-17, IL-8, and CX3CL1 secreted from human astrocytes treated with sulfatides (5 μM, 10 μM, 20 μM, 50 μM, 100 μM) for 24 h, showed a concentration-dependent increase compared to control group (Fig. 7A-D and Supplementary Fig. 1F-I). Treating astrocytes with 100 nM Olaparib for 24 h, in the presence of sulfatides, significantly decreased the release of IL-6 (Ctrl 32.8 vs. 25.3 pg/ml; 20 μM 125.4 vs. 50.6 pg/ml, *p < 0.05; 50 μM 145.3 vs. 63.0 pg/ml, **p < 0.01; Fig. 7A), IL-17 (Ctrl 7.4 vs. 10.1 pg/ml; 20 μM 78.1 vs. 35.1 pg/ml, *p < 0.05; 50 μM 105.4 vs. 54.4 pg/ml, *p < 0.05; Fig. 7B), IL-8 (Ctrl 14.9 vs. 24.5 pg/ml; 20 μM 88.0 vs. 29.3 pg/ml, *p < 0.05; 50 μM 112.1 vs. 17.9 pg/ml, *p < 0.05; Fig. 7C), and CX3CL1 (Ctrl 0.68 vs. 0.55 ng/ml; 20 μM 9.4 vs. 5.0 ng/ml, *p < 0.05; 50 μM 10.5 vs. 6.0 ng/ml, *p < 0.05; Fig. 7D). Interestingly, the overall expression of these cytokines is negatively correlated with astrocyte survival measured by MTT (Fig. 7E). Taken together, these data suggest that sulfatides exposure promotes inflammation and that Olaparib can attenuate this by inhibiting the secretion of pro-inflammatory cytokines and chemokines. Astrocytic PARP-1 overexpression may, therefore, promote CNS neuroinflammation.

### Olaparib Reduces the Chemoattraction of Human Immune Cells Towards the Soluble Microenvironment of Sulfatide-Treated Astrocytes

**Fig. 8:**
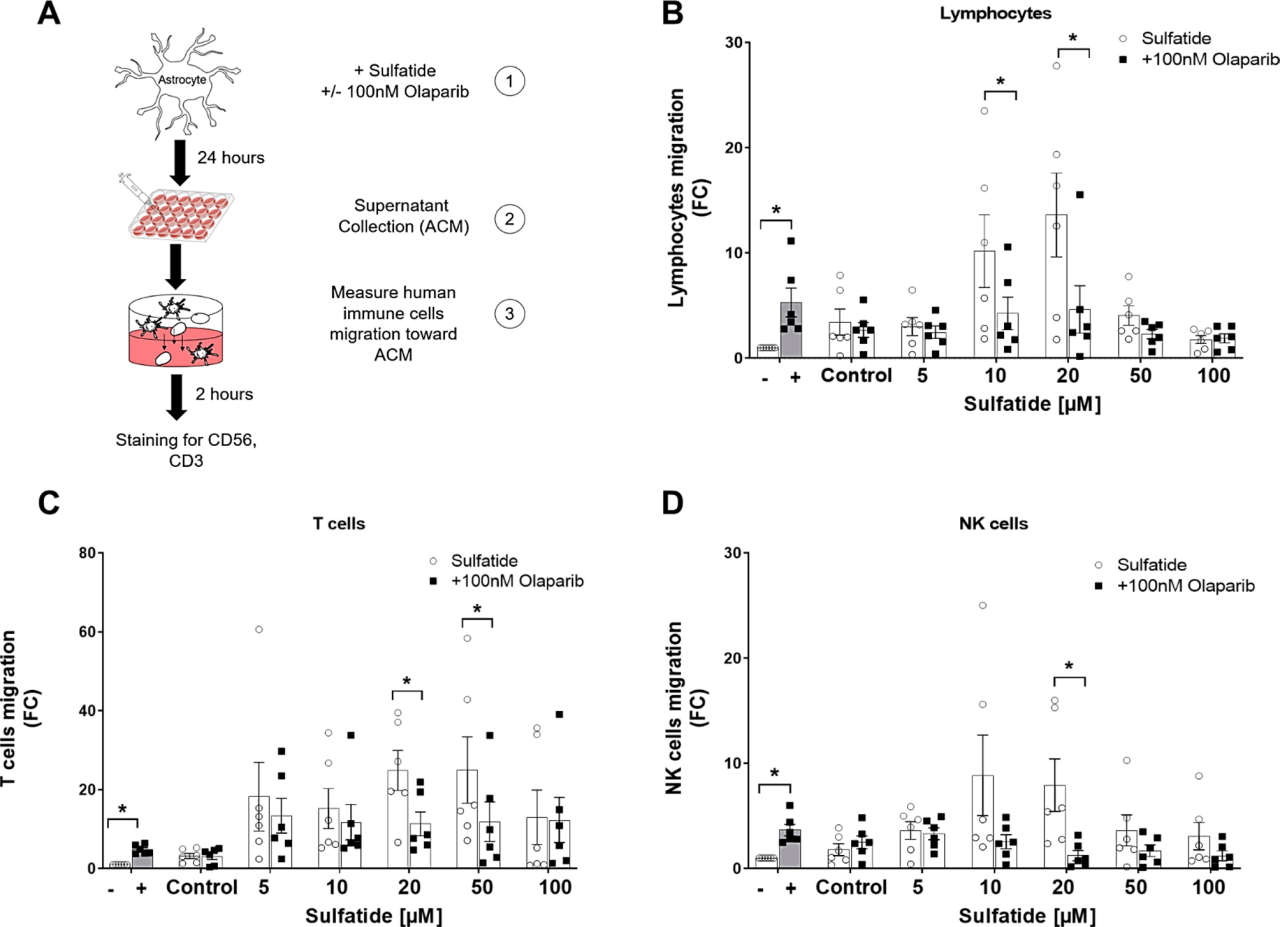
**Olaparib reduced the chemoattraction of human immune cells towards the soluble microenvironment of sulfatide-treated astrocytes.** Lymphocyte, T cell, and NK cell migration towards astrocyte-conditioned medium (ACM) from control and sulfatide-stimulated astrocytes were measured after 24 h of 100 nM Olaparib exposure. **A** Diagram of experimental timeline and treatments. **B** Bar charts showing the fold change migration of lymphocytes towards the negative control (serum-free DMEM (-)), positive control (DMEM + 20% FBS (+)), vehicle control (Control), or ACM from, astrocytes treated with sulfatides (clear circle) (5 µM, 10 µM, 20 µM, 50 µM, and 100 µM) with or without 100 nM Olaparib (black square). **C** Bar charts showing fold change migration of T cells towards the negative control (-), positive control (+), vehicle control (Control), or ACM from astrocytes treated with sulfatides (clear circle (5 µM, 10 µM, 20 µM, 50 µM, and 100 µM)) with or without 100 nM Olaparib (black square). **D** Bar charts showing FC migration of NK cells to negative control (-), positive control (+), vehicle control or ACM from astrocytes treated with sulfatides (clear circle (5 µM, 10 µM, 20 µM, 50 µM, and 100 µM)) with or without 100 nM Olaparib (black square). Data from the chemotaxis assays are presented as mean ± SEM (n = 6, using supernatants generated from MTT assay experiments). Statistical analysis was performed using a paired t-test; *p < 0.05, **p < 0.01

To elucidate the effects of sulfatide accumulation and Olaparib treatment on immune cell infiltration to the inflamed CNS, peripheral blood lymphocyte, NK cell, and T cell migration towards human astrocyte conditioned media was examined. Human astrocytes were treated, for 24 h, with sulfatides (5 μM, 10 μM, 20 μM, 50 μM, 100 μM) with or without 100 nM Olaparib. Migration of the isolated peripheral blood immune cells towards the astrocyte-conditioned medium (ACM) was measured using a transwell chemotaxis assay (Fig. 8A). Data are presented as fold-change (FC) of lymphocyte, NK cell, and T cell migration towards the lower chamber of the transwell. Data showed migration of immune cells towards the positive control (DMEM + 20% FBS) was significantly higher compared with the negative control (DMEM) demonstrating functionality of the chemotaxis system (Lymphocytes, Neg Ctrl vs Pos Ctrl: 1 vs 6.16, *p < 0.05), (T cells, Neg Ctrl vs Pos Ctrl: 1 vs 4.80, *p < 0.05), (NK cells, Neg Ctrl vs Pos Ctrl: 1 vs 3.64, *p < 0.05). The conditioned media from astrocytes treated with sulfatides increased migration of lymphocytes, T cells, and NK cells towards the lower chamber, compared to the negative control (Fig. 8B-D and Supplementary Fig. 1J-L). Importantly, conditioned medium from astrocytes treated with sulfatides and Olaparib significantly attenuated immune cell migration compared to conditioned medium from astrocytes treated with sulfatides: (Lymphocytes, 10 μM: 11.34 vs 5.01, *p < 0.05; 20 μM: 13.29 vs 5.36, *p < 0.05; Fig. 8B); (T cells, 20 μM: 24.85 vs 11.35, *p < 0.05; 50 μM: 24.94 vs 11.84, *p < 0.05; Fig. 8C); (NK cells, 20 μM: 7.92 vs 0.73, *p < 0.05; Fig. 8D). Of note, at higher concentrations of sulfatides (100 μM) there was a decrease in some cytokine release from astrocytes (IL-17 and IL-8) and immune cell migration. Together with the data from cell viability, calcium and ROS assays, it is likely that this is due to excessive sulfatide-induced astrocyte toxicity. Overall, these data suggest that the sulfatide-induced changes in pro-inflammatory response of human astrocytes are paralleled by an increased potential to recruit lymphocytes. Importantly, these changes can be attenuated by treatment with Olaparib.

## Discussion

**Fig. 9:**
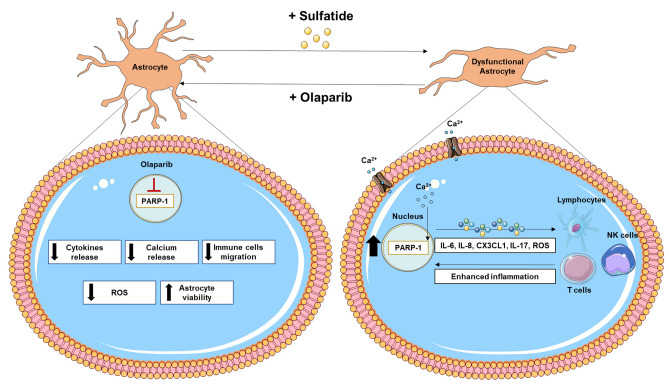
Graphical abstract. Proposed mechanism of action of Olaparib in sulfatide-treated astrocytes. Human astrocytes treated for 24 h with sulfatides increase PARP-1 expression and die. PARP-1 overexpression is modulated by Ca2+ release from the endoplasmic reticulum, thus enhancing intracellular Ca2+ concentration. PARP-1 inhibition with Olaparib reduces Ca2+ influx and cell death. Olaparib also decreases IL-6, IL-8, IL-17, and CX3CL1 release from sulfatide-stimulated astrocytes, suggesting that PARP-1 plays a role in dampening neuroinflammation in MLD. This is confirmed by the reduction of immune cell migration such as lymphocytes, NK cells, and T cells towards sulfatide-treated astrocytes. Moreover, mitochondrial stress and ROS production induced by sulfatides are rescued by PARP-1 inhibition. Future studies will focus on the signaling cascades triggered by PARP-1-mediated currents in reactive astrocytes and Olaparib as a potential therapeutic target for MLD

In this current study, we demonstrated that sulfatides caused astrocyte cell death in a concentration-dependent manner, which was attenuated by treatment with Olaparib. Olaparib showed strong efficacy in rescuing sulfatide-induced astrocyte death and rescued morphology impairment caused by treatment with sulfatides. For the first time, our data show that sulfatides induced PARP-1 activation in a concentration-dependent manner, which was inhibited by Olaparib. These data also demonstrated that sulfatides increased ROS levels and mitochondrial stress in a concentration-dependent manner in human astrocytes which were dampened by treatment with Olaparib. Olaparib also mediated the dampening of ATP-induced Ca^2+^ waves in astrocytes treated with sulfatides. Sulfatides increased pro-inflammatory cytokine secretion by human astrocytes, including IL-6, IL-8, IL-17, and CX3CL1, which could be suppressed by Olaparib. Lastly, the data showed that Olaparib dampened the enhanced chemo-attraction of peripheral blood-derived lymphocytes, NK cells and T cells toward conditioned media from human astrocytes treated with sulfatides. Taken together, this research suggests that PARP-1 plays a key role in sulfatide-induced inflammation and oxidation in human astrocytes, which can be reversed by Olaparib (Fig. 9).

Previous studies have shown that sulfatide accumulation causes neuronal dysfunction and is cytotoxic to this cell type, as well as showing that these effects could be closely linked to the pathogenesis of MLD (Molander-Melin et al. 2004). Here, we find that sulfatides induce morphological changes in cultured human astrocytes as observed by a reduction of astrocyte size, as well as cell death as measured by MTT assay. In addition, sulfatides increase ROS levels and impair mitochondrial potential suggesting that sulfatides are a source of oxidative stress for human astrocytes. Importantly, we observed that the use of Olaparib, an FDA-approved anti-cancer therapeutic, was effective in protecting astrocytes against the cytotoxic effects of sulfatides. Olaparib targets PARP-1, a transcriptional factor mainly involved in the recognition and repair of DNA single-strand breaks. PARP-1 is expressed by all brain cells in the CNS, where it contributes to multiple biological processes including cell differentiation and maturation, regulation of cholinergic and glutamatergic signaling, and memory formation. PARP-1 chronic activation, which occurs in many CNS disorders, is responsible for augmented oxidative stress and cell death via apoptotic-independent pathways. Our findings showed sulfatides to cause PARP-1 overexpression and reversal by Olaparib. In sulfatide-treated astrocytes, we found that Olaparib augmented cell survival and reduced ROS production and mitochondrial damage. Overall, this set of data supports PARP-1 being a key driver of sulfatide-induced astrocyte damage and which can be effectively targeted by Olaparib to reverse sulfatide toxicity.

Under physiological conditions, calcium signaling plays a critical role in communication between astrocytes and neurons, while dysregulated cellular calcium contributes to pathophysiological conditions such as necrosis, apoptosis, autophagy deficits, and neurodegeneration (Bezprozvanny 2009). Furthermore, calcium is central to many astrocytic signaling events, including mitogenic responses to growth factors, gene expression, release of signaling molecules, and responses to neurotransmitters such as ATP and glutamate (Bezzi et al. 1998). The downstream pathways essential for the execution of cell death in response to PARP-1-mediated metabolic alterations remain poorly understood, but there are some evidence that support the involvement of aberrant calcium signaling. The regulatory mechanisms controlling PARP-1 function to either promote cell survival or cell death in response to DNA damage also remain enigmatic. PARP-1 facilitates DNA repair and cell survival in response to a variety of DNA-damaging agents. PARP-1 also mediates programmed necrosis (Zong et al. 2004), as well as caspase-independent apoptotic cell death following severe levels of DNA damage (Yu et al. 2002). Given that we found that sulfatides promoted the expression of PARP-1 and also increase the levels of intracellular calcium, and that these effects are dampened by Olaparib, we consider a coupling between expression of PARP-1 and calcium signaling, which play a role in astrocyte death. We note, however, that the temporal order of these events remains to be elucidated.

In the past, PARP-1 was thought to contribute to disease pathogenesis primarily by inducing cell death (Szabó 2002). However, more recently it has been recognized that PARP-1 activity may also modulate the transcription and translation of genes involved in inflammation (Szabó 2002; Kraus and Lis 2003). In the majority of CNS disorders, inflammatory processes trigger changes in BBB function that are central to the infiltration of immune cells into CNS tissues, inflammatory responses and, ultimately, the pathogenesis of the disease (Hooper et al. 2000; Kean et al. 2000). Inflammatory processes have also been implicated in the pathogenesis of MLD while the overexpression of pro-inflammatory cytokines has been reported in the brains of MLD patients (Thibert et al. 2016). Since astrocytes are an important source of such neuromodulatory cytokine and chemokine production (Lieberman et al. 1989; Rothhammer and Quintana 2015), the effects of sulfatides treatment on astrocyte secretion of pro-inflammatory cytokines, IL-17, IL-8, IL-6, and CX3CL1, were examined. Our data show that sulfatides increased pro-inflammatory cytokine release from sulfatide-stimulated human astrocytes while Olaparib treatment significantly attenuated this effect. Therefore, PARP-1 inhibition may cause a general dampening of pro-inflammatory cytokine secretion in astrocytes.

In some disease models, such as multiple sclerosis, PARP-1 activity has been proposed to alter leukocyte migration by modifying the expression of adhesion molecules (Zingarelli et al. 1998; Sharp et al. 2001). In agreement with these findings, our data show that Olaparib treatment reduced human peripheral blood immune cell migration towards the soluble microenvironment (i.e., astrocyte-conditioned media) of sulfatide-treated human astrocytes. The Olaparib-induced decreases in human lymphocyte, NK cell and T cell migration were positively correlated with the Olaparib-mediated reductions in pro-inflammatory mediators implicated in the disease pathogenesis and with an increased astrocyte survival rate. These studies go some way in translating the findings to a human setting showing that sulfatide-treated human astrocytes promote migration of immune cells, that were obtained from the peripheral blood of healthy humans.

## Conclusion

Metachromatic leukodystrophy (MLD) is a fatal leukodystrophy with no current curative treatment. Therefore, the need for new drugs and pharmacological approaches that can cure or improve survival and/or quality of life for these patients is urgent. Accumulation of sulfatides, propagation of pro-inflammatory cytokines, demyelination, and the widespread loss of oligodendrocytes are all hallmarks of MLD. To date, little is known about the role of astrocytes in MLD, whereas the majority of studies have mainly focused on the role of oligodendrocytes in this illness. Altered astrocytic function has gained recognition as a major contributing factor to a growing number of neurological disorders (Claycomb et al. 2013) and the belief that astrocytic dysfunction significantly contributes to the development of inflammation in the CNS has gained traction in the past number of years (Sharma et al. 2010). In addition, the immunomodulatory functions of astrocytes are now being shown to actively participate in the pathogenesis of several demyelinating disorders (Sharma et al. 2010). It has also been reported that astrocytic processes may closely surround demyelinating fibres and that astrocytes release a number of factors to promote myelination. This might support the idea that demyelination in MLD may not be solely attributed to oligodendrocyte dysfunction but involve a central role for astrocytes. Thus, while astrocytic reactivity in MLD may represent a secondary response to demyelination, these cells may be primary responders to sulfatides and contribute to the pathogenesis of MLD.

Here we demonstrated that sulfatides treatment alters human astrocyte cell survival, mitochondrial function, ROS levels, calcium signaling, and pro-inflammatory cytokine levels. Our data also indicate that sulfatides induce overactivation of PARP-1 in human astrocytes. Importantly, we show sulfatide-treated human astrocytes promote migration of human peripheral blood immune cells. All these effects of sulfatides were attenuated by the PARP-1 inhibitor Olaparib (Fig. 9). Overall, our study translates somewhat into a human cell biology setting and suggests that PARP-1 inhibition may be a therapeutic option in MLD, where marketed drugs such as Olaparib may be worthy of further investigation.

### Supplementary Information

Below is the link to the electronic supplementary material.

Supplementary file1: **Control experimental data for effects of sulfatides.** (**A**-**B**) **Sulfatides increase vimentin’s expression in human astrocytes**. Human astrocytes were treated with sulfatides (5 µM, 10 µM, 20 µM, 50 µM, and 100 µM for 24 hours). Confocal images were captured at ×20 and a number of 15 images were analyzed per condition. (**A**) Bar graph illustrating changes in astrocytes area after the treatment with sulfatides (5 µM, 10 µM, 20 µM, 50 µM, and 100 µM). (**B**) Bar graph illustrating changes in the intensity of vimentin fluorescence after the treatment with sulfatides (5 µM, 10 µM, 20 µM, 50 µM, and 100 µM). Data are presented as mean ± SEM (n = 5), one-way ANOVA following by Dunnett post hoc test, *p<0.05, **p<0.01, ***p<0.001 compared to control group. (**C**-**D**) **Sulfatides induce PARP-1 activation and expression in human astrocytes**. Confocal images were captured at ×40 or ×63 magnification and a number of 15 images were analyzed per condition. Treatment with sulfatides induces PARP-1 translocation to nuclei and PAR expression (5 µM, 10 µM, 20 µM, 50 µM, and 100 µM). Semi-quantitative analysis of PARP-1 or PAR-associated fluorescence in the nuclei of astrocytes. Data are presented as mean ± SEM (n = 5), one-way ANOVA followed by Turkey’s post hoc test, *p<0.05, **p<0.01, ***p<0.001 compared to control group. (**E**-**F**) **Sulfatides increase reactive oxygen species (ROS) generation in human astrocytes and cause mitochondrial stress**. (**E**) Quantification of ROS levels was determined by 2’-7’ dichlorofluorescein diacetate (DCFH-DA) staining in human astrocytes treated with 5 µM, 10 µM, 20 µM, 50 µM, and 100 µM of sulfatides. Data are presented as the mean absorbance levels (at 490 nm). (**F**) To confirm that PARP-1 activation had negligible effects on cellular stress, the JC-1 assay was performed to measure changes in the mitochondrial membrane potential of sulfatides (5 µM, 10 µM, 20 µM, 50 µM, and 100 µM). Data are presented as the mean absorbance levels (at 570 nm). The measures of fluorescence signal are reported in the graphs as fold change (FC) or percentage of each condition versus the control and presented as mean ± SD (n = 5). Statistical significance was determined by paired t-test, ***p<0.001. (**G**-**J**) **Sulfatides increase cytokine and chemokine release from human astrocytes**. Cytokine and chemokine release from control and sulfatide-stimulated astrocytes (clear circle) were measured after 24 hours. Sulfatides (5 µM, 10 µM, 20 µM, 50 µM, and 100 µM) showed a significant increase in (**G**) IL-6, (**H**) IL-17, (b) IL-8, and (**J**) CX3CL1 release into the cell culture medium, as measured by ELISA. (Data from the ELISA assays are presented as mean ± SEM. IL-6, IL-17, IL-8, and CX3CL1 ELISA's were repeated n = 5 (using supernatants generated from MTT assay experiments). Statistical analysis was performed using a paired t-test; *p<0.05, **p<0.01, ***p<0.001. (**K**-**M**) **Sulfatides induce the chemoattraction of human immune cells towards the soluble microenvironment of astrocytes**. Lymphocyte, T cell, and NK cell migration towards astrocyte-conditioned medium (ACM) from control and sulfatide-stimulated astrocytes were measured after 24 hours. (**K**) Bar charts showing the fold change (FC) migration of lymphocytes towards the negative control (serum-free DMEM (-)), positive control (DMEM+20% FBS (+)), vehicle control (Control), or ACM from, astrocytes treated with sulfatides (5 µM, 10 µM, 20 µM, 50 µM, and 100 µM). (**L**) Bar charts showing FC migration of T cells towards the negative control (serum-free DMEM (-)), positive control (DMEM+20% FBS (+)), vehicle control (Control), or ACM from astrocytes treated with sulfatides (5 µM, 10 µM, 20 µM, 50 µM, and 100 µM). (**M**) Bar charts showing FC migration of NK cells to negative control (-), positive control (+), vehicle control, and ACM following treatment of sulfatides (5 µM, 10 µM, 20 µM, 50 µM, and 100 µM). Data from the chemotaxis assays are presented as mean ± SEM (n = 6, using supernatants generated from MTT assay experiments). Statistical analysis was performed using a paired t-test; *p<0.05, **p<0.01 compared to negative control (JPG 815 KB)

## Data Availability

The datasets and materials generated during and/or analysed during the current study are available from the corresponding author on reasonable request.
