# Supplementary figures and images for: Elucidating the Therapeutic Utility of Olaparib in Sulfatide-Induced Human Astrocyte Toxicity and Neuroinflammation
Source: J Neuroimmune Pharmacol. 2023 Nov 4;18(4):592–609. doi: 10.1007/s11481-023-10092-9 (PMC10770269; doi:10.1007/s11481-023-10092-9)

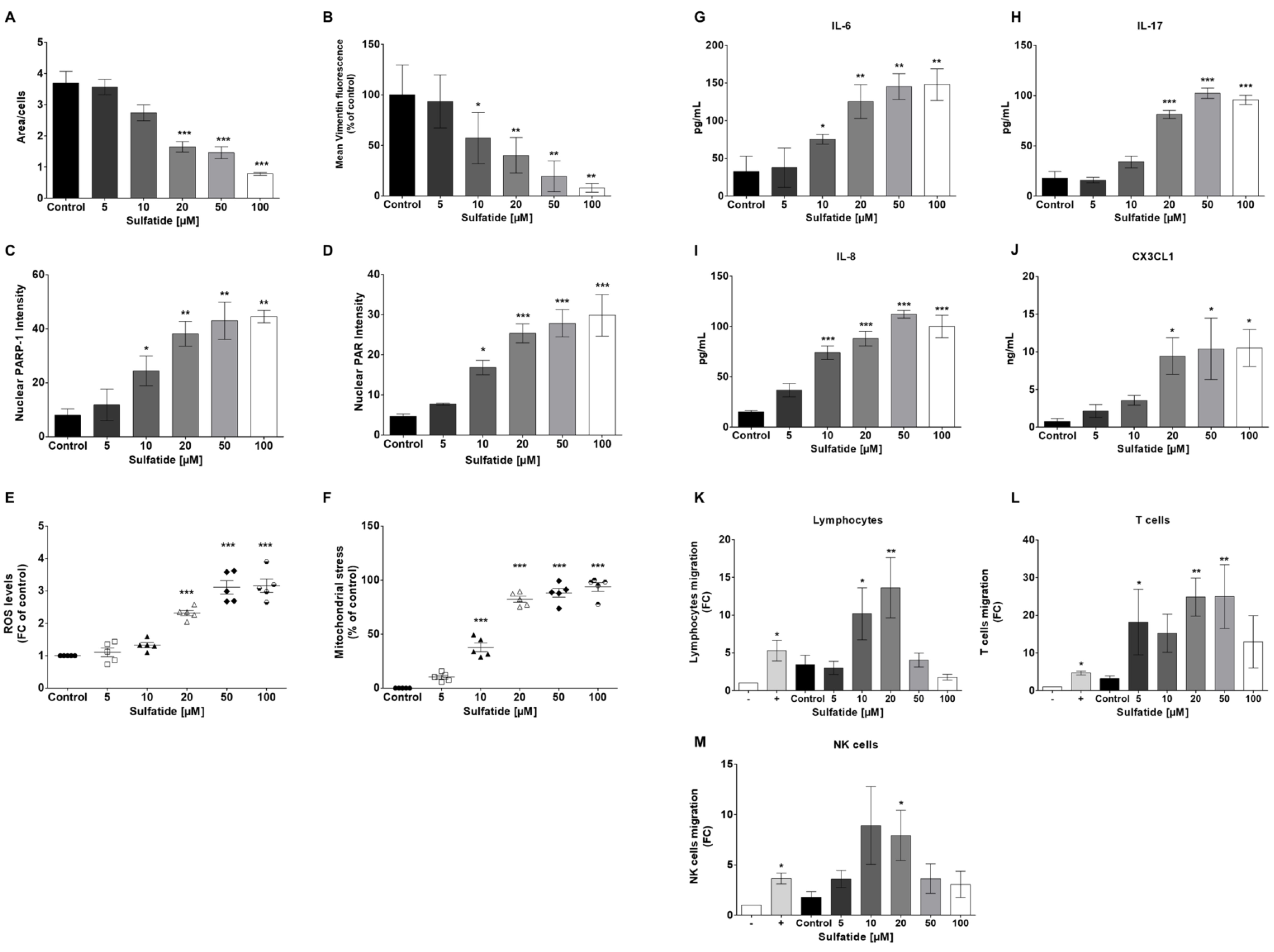

Supplement: Supplementary file 1 — Supplementary file1: Control experimental data for effects of sulfatides. (A-B) Sulfatides increase vimentin’s expression in human astrocytes. Human astrocytes were treated with sulfatides (5 µM, 10 µM, 20 µM, 50 µM, and 100 µM for 24 hours). Confocal images were captured at ×20 and a number of 15 images were analyzed per condition. (A) Bar graph illustrating changes in astrocytes area after the treatment with sulfatides (5 µM, 10 µM, 20 µM, 50 µM, and 100 µM). (B) Bar graph illustrating changes in the intensity of vimentin fluorescence after the treatment with sulfatides (5 µM, 10 µM, 20 µM, 50 µM, and 100 µM). Data are presented as mean ± SEM (n = 5), one-way ANOVA following by Dunnett post hoc test, *p<0.05, **p<0.01, ***p<0.001 compared to control group. (C-D) Sulfatides induce PARP-1 activation and expression in human astrocytes. Confocal images were captured at ×40 or ×63 magnification and a number of 15 images were analyzed per condition. Treatment with sulfatides induces PARP-1 translocation to nuclei and PAR expression (5 µM, 10 µM, 20 µM, 50 µM, and 100 µM). Semi-quantitative analysis of PARP-1 or PAR-associated fluorescence in the nuclei of astrocytes. Data are presented as mean ± SEM (n = 5), one-way ANOVA followed by Turkey’s post hoc test, *p<0.05, **p<0.01, ***p<0.001 compared to control group. (E-F) Sulfatides increase reactive oxygen species (ROS) generation in human astrocytes and cause mitochondrial stress. (E) Quantification of ROS levels was determined by 2’-7’ dichlorofluorescein diacetate (DCFH-DA) staining in human astrocytes treated with 5 µM, 10 µM, 20 µM, 50 µM, and 100 µM of sulfatides. Data are presented as the mean absorbance levels (at 490 nm). (F) To confirm that PARP-1 activation had negligible effects on cellular stress, the JC-1 assay was performed to measure changes in the mitochondrial membrane potential of sulfatides (5 µM, 10 µM, 20 µM, 50 µM, and 100 µM). Data are presented as the mean absorbance levels (at 570 nm). The measur [file 11481_2023_10092_MOESM1_ESM.jpg]
